# Comparative analysis of solvent-based and solvent-free (melting) methods for fabricating 3D-printed polycaprolactone-hydroxyapatite composite bone scaffolds: physicochemical/mechanical analyses and *in vitro* cytocompatibility

**DOI:** 10.3389/fbioe.2024.1473777

**Published:** 2025-01-06

**Authors:** Brigita De Vega, Abir Dutta, Aisha Mumtaz, Bob C. Schroeder, Craig Gerrand, Ashleigh S. Boyd, Deepak M. Kalaskar

**Affiliations:** ^1^ Division of Surgery and Interventional Science, University College London, Royal Free Hospital Campus, London, United Kingdom; ^2^ Institute of Orthopaedics and Musculoskeletal Science (IOMS), Division of Surgery and Interventional Science, University College London, Stanmore, United Kingdom; ^3^ Department of Mechanical Engineering, Indian Institute of Technology Tirupati, Andhra Pradesh, India; ^4^ Department of Chemistry, University College London, London, United Kingdom; ^5^ Bone and Soft Tissue Tumour Service, Royal National Orthopaedic Hospital, Stanmore, United Kingdom; ^6^ UCL Institute of Immunity and Transplantation, Pears Building, London, United Kingdom

**Keywords:** composite, bone scaffold, 3D printing, additive manufacturing, polycaprolactone, hydroxyapatite

## Abstract

**Purpose:**

The study conducts a comparative analysis between two prominent methods for fabricating composites for bone scaffolds—the (solid) solvent method and the solvent-free (melting) method. While previous research has explored these methods individually, this study provides a direct comparison of their outcomes in terms of physicochemical properties, cytocompatibility, and mechanical strength. We also analyse their workflow and scalability potentials.

**Design/methodology/approach:**

Polycaprolactone (PCL) and hydroxyapatite (HA) composites were prepared using solvent (chloroform) and melting (180°C) methods, then 3D-printed using an extrusion-based 3D printer to fabricate scaffolds (8 × 8 × 4 mm). Rheology, scanning electron microscopy (SEM), energy-dispersive X-ray spectroscopy (EDX), Fourier transform infrared spectroscopy (FTIR), X-ray diffraction (XRD), thermogravimetric analysis (TGA), differential scanning calorimetry (DSC), accelerated degradation, mechanical/compression test, wettability/contact angle, live/dead assay, and DNA quantification (Picogreen) assays were evaluated.

**Findings:**

The study finds that scaffolds made via the solid solvent method have higher mechanical strength and degradation rate as compared to those from the melting method, while both methods ensure adequate cytocompatibility and homogenous hydroxyapatite distribution, supporting their use in bone tissue engineering.

**Originality:**

This research investigates the utility of chloroform as a solvent for PCL composite in a direct comparison with the melting method. It also highlights the differences in workflows between the two methods and their scalability implications, emphasizing the importance of considering workflow efficiency and the potential for automation in scaffold fabrication processes for bone tissue engineering applications.

## 1 Introduction

Biomaterials such as polycaprolactone (PCL), polylactic acid (PLA), and poly (lactic-co-glycolic) acid (PLGA) are frequently employed in 3D printing bone scaffolds. However, their limited bioactivity has spurred the development of composite materials. By blending these thermoplastic polymers with calcium-phosphate-based ceramics like hydroxyapatite (HA) and tricalcium phosphate (TCP), researchers aim to enhance the biological properties of scaffolds and mimic native bone structure (Turnbull et al., 2018; Wang et al., 2021; Santos Beato et al., 2023). Encouraging clinical outcomes have been observed in Germany, Australia, and South Korea, where 3D printed bone scaffolds made from composite materials have successfully treated complex massive bone defects, some reaching lengths of up to 36 cm. Most patients experienced bone regeneration and reported improved quality of life at 6–12 months follow-up (Budharaju et al., 2023; Castrisos et al., 2022; Jeong et al., 2022; Kobbe et al., 2020; Laubach et al., 2022).

Although design and fabrication have been well explored, the influence of preparation methods of composite materials on the properties of printed scaffolds is not well understood. Zimmerling et al. explored four PCL-HA composite preparation methods (melt-blending, powder blending, liquid solvent, and solid-solvent) and concluded that melt-blending appears to be the most favourable in terms of printability, mechanical properties, and efficiency (Zimmerling et al., 2021). However, solvent-based methods, including liquid solvent and solid solvent, remain the most popular despite concerns about solvent residue and poor shape fidelity associated with liquid solvent methods (Huang et al., 2022).

In the solid-solvent method, the solvent fully evaporates before printing, resulting in similar shape fidelity to the melt-blending method (Zimmerling et al., 2021). Biscaia et al. compared the melt-blending method with the solid-solvent method using dimethylformamide (DMF) to mix PCL and HA, showing that the melt-blending method is superior in terms of mechanical and biological properties (Biscaia et al., 2022). Nonetheless, the choice of solvent in composite preparation can significantly influence the resultant mixture and its properties, necessitating exploration of different solvents and their potential effects (Patlolla and Arinzeh, 2014; Zhang et al., 2019; Altun et al., 2022).

Material processing involving chemical solvents or heating (as in the melt-blending method) may affect material properties, the uniformity of the composite ink, the printing process, and the final products (Yan and Awad, 2023; Peng et al., 2024). Additionally, the two methods have distinct workflows that may appeal to different stakeholders depending on the production scale and application. Therefore, this study aims to conduct a comparative analysis between two methods for fabricating bone scaffolds—the solid-solvent method and the solvent-free (melting) method. While previous research has explored these methods individually, this study provides a direct comparison of their outcomes in terms of physicochemical properties, cytocompatibility, and mechanical strength.

## 2 Materials and methods

### 2.1 Preparation of 3D printing ink using solvent and solvent-free (melting) methods

**FIGURE 1:**
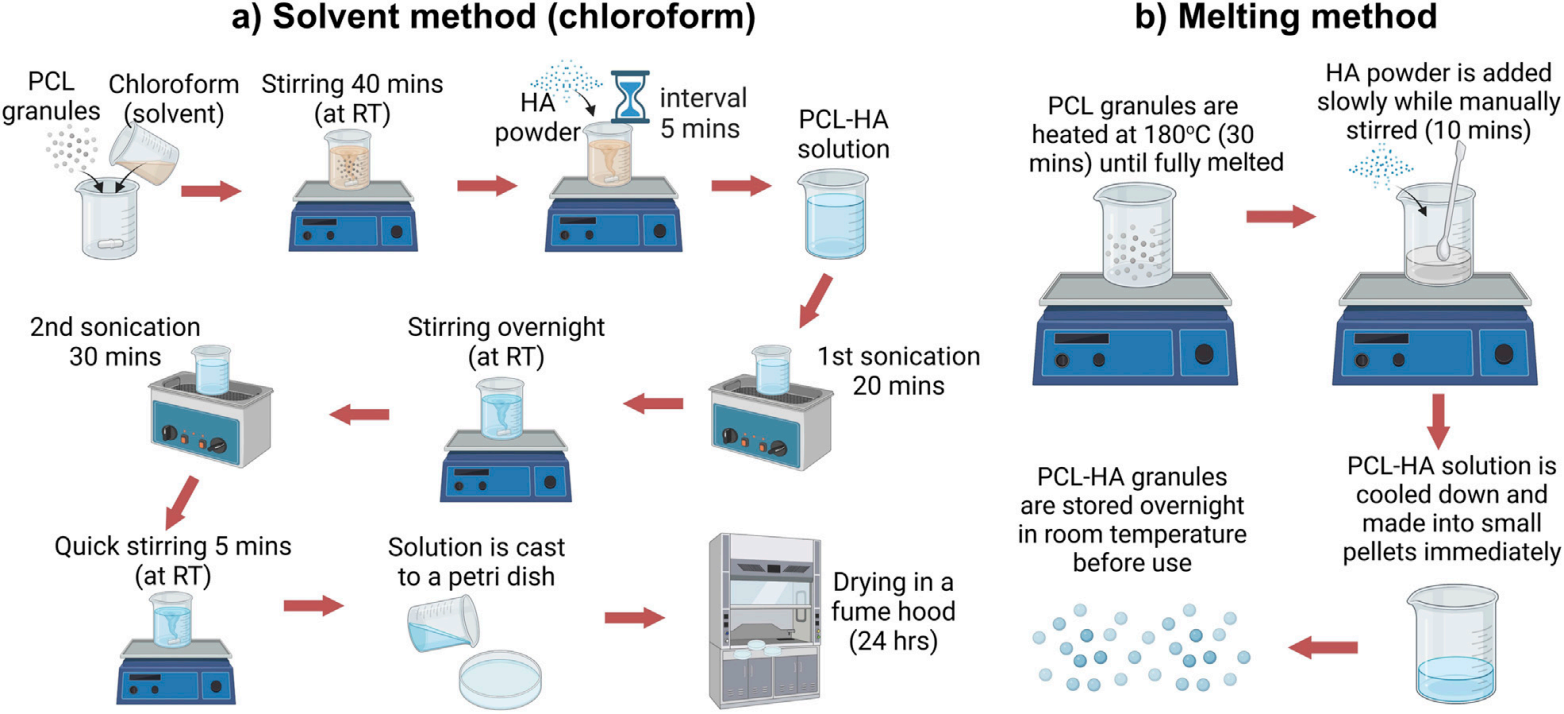
PCL-HA composite preparation using solid solvent method **(A)** and solvent-free/melting method **(B)**. Created with BioRender.com (licence number CP26VLLARI).

Polycaprolactone (PCL) granules CAPA 6500D (M_w_ 50,000) (Ingevity, United Kingdom) and medical-grade hydroxyapatite (HA) powder (<20 μm, obtained from Ceramisys Ltd., United Kingdom) were mixed at 90:10 ratio (w/w %) using two methods, i.e., solid-solvent method (hereafter referred to as the solvent method) and melting (solvent-free) method (Figure 1).

#### 2.1.1 Solvent method

The solvent method (SM) was established based on a modification from an existing protocol (Figure 1A) (Abdal-hay et al., 2018; Doyle et al., 2021). PCL granules were dissolved in chloroform (CHCl_3;_ Sigma-Aldrich, United Kingdom) at 15% w/v, followed by stirring using a magnetic stirrer for 40 min. Next, HA was added in several batches and stirred for 5 min in between the batches to avoid agglomeration. The solution was then sonicated for 20 min (1st sonication), followed by an overnight stirring (12 h). The next day, the solution was again sonicated for 30 min (2nd sonication) to eliminate air bubbles, followed by a quick stirring for 5 min. The solution was cast in a petri dish and placed under a fume hood for 24 h. Finally, the dried PCL-HA (9:1) composite was cut into small pieces (approximately 4 × 4 mm) (Supplementary Appendix S1).

#### 2.1.2 Melting method

In the solvent-free (melting) method (MM), PCL granules were heated at 180°C for 30 min. HA powder was slowly poured into the melted PCL and stirred manually for 10 min until homogenous. PCL-HA composite was cooled down and made into pellets immediately (diameter 3–4 mm) (Supplementary Appendix S1). Lastly, PCL-HA pellets were stored in a desiccator overnight at room temperature before use (Figure 1B).

### 2.2 Rheology, extrusion-based 3D printing and optimisation of printing parameters

#### 2.2.1 Rheology of PCL-HA composite prepared using solvent and solvent-free (melting) methods

Rheological characterization was performed using Kinexus Prime pro + Rheometer (NETZSCH, Germany) equipped with a 20 mm parallel plate setup, with a 500 μm gap. An amplitude sweep test was carried out, ranging from 10^−2^–10^4^% strain, at a frequency of 1 Hz to assess the material’s viscoelastic properties. Before measurements, a heating time of 60 min was performed to simulate the 3D printing pre-heating condition. The samples were measured at 80, 100, and 120°C based on our preliminary study regarding the working printing temperature of our material.

#### 2.2.2 Extrusion-based 3D printing and optimisation of printing parameters

**FIGURE 2:**
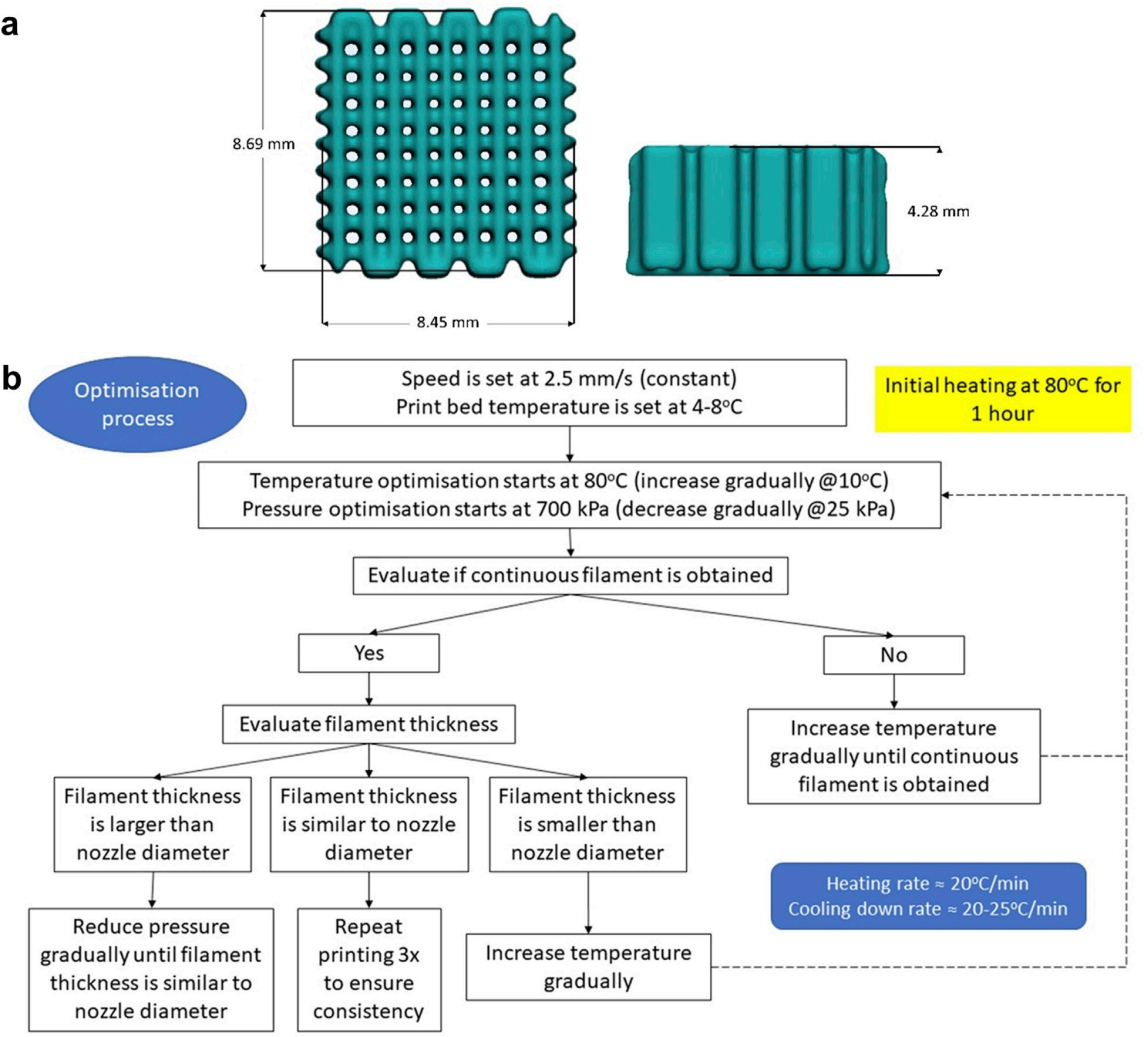
**(A)**: Scaffold design in STL (Standard Tessellation Language) file format (length × width × height = 8.45 × 8.69 × 4.28 mm): **(B)**: Schematic showing the optimisation of the printing process and parameters.

SolidWorks 2021 (Dassault Systèmes, United States) was used to design a cuboid with a dimension of 10 × 10 × 4 mm. STL (Standard Tessellation Language) file was transferred to an extrusion based BIOX6 bioprinter (Cellink, Sweden) and processed with the bioprinter’s slicing software (DNA Studio 4, BICO, Sweden) to produce 90% infill density (10% porosity) and have the external perimeter removed (Figure 2A). Hence, the final dimension of the scaffold design was ∼8 × 8 × 4 mm (after external perimeter removal). The optimisation of printing parameters is presented in Figure 2B. A thermoplastic printhead (heating capacity up to 250°C) containing a metal cartridge was filled with the material and pre-heated at 80°C for an hour to allow the material to fully melt. A standard metal nozzle size (0.4 mm) was used in all experiments. Printing speed was constant at 2.5 mm s^−1^ while print bed temperature was set at 4°C–8°C.

Then, the optimisation process started at 80°C and 700 kPa (the highest pressure the machine is capable of). The optimal printing temperature was determined as the lowest temperature needed to produce a continuous filament with adequate thickness (similar to nozzle diameter). Meanwhile, the optimal pressure was defined as the minimum pressure applied to obtain a continuous filament with adequate thickness. Each time the temperature was increased, a waiting time of 15 min was implemented before starting the next print. Filament thickness/width was evaluated visually. When the filament thickness obtained was found to be similar to the nozzle diameter, the printing was repeated thrice to ensure repeatability (Figure 2B).

### 2.3 Physicochemical characterisation of 3D-printed bone scaffolds

#### 2.3.1 Measurement of the scaffolds

The dimensions (length × width × height) of the 3D printed scaffolds (n = 4) were measured using a digital calliper and compared with designed file dimensions.

#### 2.3.2 Scanning electron microscopy (SEM)

Scanning Electron Microscopy (Zeiss Field Emission SEM Sigma 300 VP, United Kingdom) was performed to analyse the scaffolds’ surface morphology. Scaffolds (n = 4) were sputter-coated with gold/palladium for 1 min at 20 mA. Images were captured at electron high tension (EHT) of 3–10 kV and an aperture of 30 μm. Filament width and pore size were measured at four random areas and averaged (n = 4).

#### 2.3.3 Energy-dispersive X-ray spectroscopy (EDX)

Elements microanalysis was assessed using Zeiss SmartEDX (Zeiss, United Kingdom), to observe HA particles distribution within PCL filaments (by assessing Ca and P elements) at ×50 magnification (30 kV, aperture 60 μm). EDX spectra are obtained by bombarding a sample with high-energy electrons, which induce characteristic X-ray emission from the sample’s atoms, detected by an X-ray detector and finally the signals are converted into a spectrum. Zeiss SmartEDX Standard software v1.3.1 was employed to perform surface mapping and analyse the spectra. Analyses were performed in the centre of the scaffolds (n = 4).

#### 2.3.4 Fourier transform infrared spectroscopy (FTIR)

FTIR was carried out to (1) assess whether the solvent method leaves any residue, and (2) identify specific chemical bonds of biomaterials. A small piece of the material was cut (2 × 2 mm) and loaded in Spectrum Two FT-IR Spectrometer (Perkin Elmer, United States), wavelength ∼4,000–400 cm^−1^.

#### 2.3.5 X-ray diffraction analysis (XRD)

X-ray diffraction analysis was performed on the samples to understand the influence of processing methods on the different phases present. XRD spectra were acquired in flat plate geometry with nickel-filtered copper Kα radiation using a Bruker D8 advance diffractometer (Bruker, Coventry, United Kingdom). Data were collected using a Lynx eye detector with an incident slit of 0.2 mm and step size of 0.019° over a 2θ range of 10°–100°.

#### 2.3.6 Thermogravimetric analysis (TGA)

Thermal gravimetric analysis (TGA) was carried out on a TGA 5500 (TA Instruments, United States). Approximately 3 mg of each sample was weighed into a high temperature TA platinum pan, before being heated at 10°C min^−1^ from 50°C to 700°C under a nitrogen atmosphere. The onset of degradation was obtained from the intersection of two tangents.

#### 2.3.7 Differential scanning calorimetry (DSC)

Differential scanning calorimetry (DSC) was carried out on a TA Instruments DSC 2,500 with TA LN2P liquid nitrogen pump attached by cycling between −100°C and 100°C at a rate of 10°C min^−1^ under a helium atmosphere. Between 4 and 6 mg of each material was weighed out on a 6 d. p. balance (Sartorius Quintix 35-1S, United Kingdom) into TA Tzero aluminium pans. The glass transition temperatures (T_g_) were extracted by heating the sample at a rate of 20°C min^−1.^

#### 2.3.8 Accelerated degradation test

Accelerated degradation tests were conducted following a previously established protocol (Huang et al., 2022; Daskalakis et al., 2023) to investigate the hydrolytic degradation process of PCL-based scaffolds. 3D printed scaffolds (n = 4) were initially weighed using a high-precision balance, and then immersed in a 5 M NaOH solution (1 mL) at 37°C for 5 days. Each day, four samples from each group were removed from the NaOH solution, rinsed three times with deionized water, and left to dry overnight in a fume hood. Once fully dried, the samples were re-weighed to determine the extent of weight reduction. To ensure accuracy, each weight measurements (both initial weight and final weight) were conducted three times, then the values were averaged and compared. SEM imaging was also conducted (for samples at day-1 and day-5) to show the morphological changes due to hydrolytic degradation process.

#### 2.3.9 Mechanical (compression) test

Compression tests on the scaffolds were conducted according to the ASTM D695 (ASTM International, 2016). For compression test only, the scaffolds were printed to a dimension of ∼8 × 8 × 16 mm (width to height ratio 1:2). Measurements were carried out using ZwickRoell Z005 universal testing machine (ZwickRoell, Austria) with a maximum load set at 5 kN and crosshead speed 5 mm/min (n = 4). The load was applied until the scaffold fractured, with no preload applied. Prior to compression testing, the exact dimensions of scaffolds were measured (length × width × height) using a digital calliper. Maximum load (upper yield point) and displacement at upper yield point data were collected from the testing machine. Yield strength (Equation 1), yield strain (Equation 2), and Young’s modulus (Equation 3) were calculated based on the following formulas:

σ=F/A (1)

where

σ = Yield strength/maximum stress (MPa)

F = Maximum resistance force/upper yield point (N).

A = Surface area (mm^2^)

ɛ=ΔL/L (2)

where

ɛ = Strain

ΔL = Displacement at maximum load/upper yield point (mm)

L = gauge length (mm)

E=σ/ɛ (3)

where

$$E=\text{Young}’s\text{ modulus }\left(\text{MPa}\right)$$

#### 2.3.10 Wettability (contact angle) test

Cast flat surface samples were prepared to perform the wettability (water contact angle) test (4 μL water drop) using Krüss Drop Shape Analyzer DSA25E (Krüss, Germany). Samples were measured in four random places and averaged (n = 4).

### 2.4 Cytocompatibility assessment of 3D-printed bone scaffolds

Human Saos-2 cell line passage 25–27 (Sigma-Aldrich, United Kingdom) was expanded in T75 flasks. Dulbecco’s Modified Eagle Medium (DMEM) (Thermofisher, United Kingdom), 10% foetal bovine serum (FBS) (Thermofisher, United Kingdom), and 1% penicillin/streptomycin (Sigma-Aldrich, United Kingdom) were used as culture media. Media was changed every 3 days. Prior to cell seeding, scaffolds were immersed in 70% ethanol and left overnight under Ultraviolet (UV) light. The next day, sterilised scaffolds were washed with PBS (Thermofisher, United Kingdom) 3 times, put in sterile 24-well plates inside the class II biosafety cabinet (BSC), immersed in culture media, and incubated for 48 h (37°C, 5% CO_2_). Thereafter, 50,000 cells in 50 μL culture media were seeded (seeding density 0.2 × 10^6^ cells mL^−1^ scaffold) (De Luca et al., 2018) on top of the scaffolds (static method), and samples (seeded scaffolds) were put in an incubator for 1 hour to allow cell attachment. Next, samples were moved into new 24-well plates, and 1 mL culture media was added to each well.

#### 2.4.1 Live and dead assay

Live and dead assay staining (Invitrogen L3224, Thermofisher, United Kingdom) were performed on days 1 and 7 to evaluate cell viability qualitatively. Live/dead assay was performed as per the manufacturer’s protocol. After washing with PBS, scaffolds were immersed in 1 mL staining solution. The staining solution was obtained by adding 2 μL red-fluorescent ethidium homodimer-1 and 0.5 μL green, fluorescent calcein-AM per mL PBS. After incubation for 25 min (37°C, 5% CO_2_) followed by washing with PBS, samples were examined under a fluorescent microscope (Olympus BX63F, Olympus, United Kingdom). Images were captured at ×4 and ×10 magnification.

#### 2.4.2 Picogreen assay

Double-stranded DNA (dsDNA) was quantified using Quant-iT™ PicoGreen™ dsDNA Reagent and Kit (Invitrogen P11496, Thermofisher, United Kingdom) according to the manufacturer’s protocol. Firstly, a lysis buffer containing 1% Triton X in 1X TE (10 mM Tris-HCl, 1 mM EDTA) buffer was prepared. Triton X-100 (Sigma-Aldrich, United Kingdom) was diluted 100-fold to obtain Triton X solution, while 20X TE buffer (that came with the Picogreen kit) was diluted 20-fold to obtain 1X TE buffer (UltraPure™ DNase/RNase-Free Distilled Water was used for the dilution).

Next, samples (n = 4) were washed two times in PBS, immersed in the lysis buffer, and incubated (37°C, 5% CO_2_) for 45 min. Samples underwent freeze-thaw cycles three times (45 min each) for further cell lysis and to release their DNAs. Then, the Picogreen working solution was prepared by diluting Picogreen reagent 200-fold in 1X TE buffer. Finally, 100 μL of samples’ solution and 100 μL of Picogreen working solution were added to a black 96-well plate and incubated for 5 min (protected from light) before being read in a microplate reader at 480 nm excitation, 520 nm emission (TECAN Infinite 200, Switzerland). Lambda DNA standard curve was used as a reference to calculate the amount of DNA.

### 2.5 Statistical analysis

Continuous data were presented in mean ± SD. Normality tests were performed using QQ-plot and Shapiro-Wilk tests. Normally distributed data were analysed using parametric one-way ANOVA test (>2 groups) or independent *t*-test (2 groups). In comparison, non-normally distributed data were analysed using non-parametric Kruskal–Wallis test (>2 groups) or Mann-Whitney test. The *p*-value significance level was set to 0.05. Tukey *post hoc* test was also performed when appropriate. All statistical analyses were performed using GraphPad Prism 9 (Dotmatics, United States).

## 3 Results

### 3.1 Rheology and printing optimisation

**FIGURE 3:**
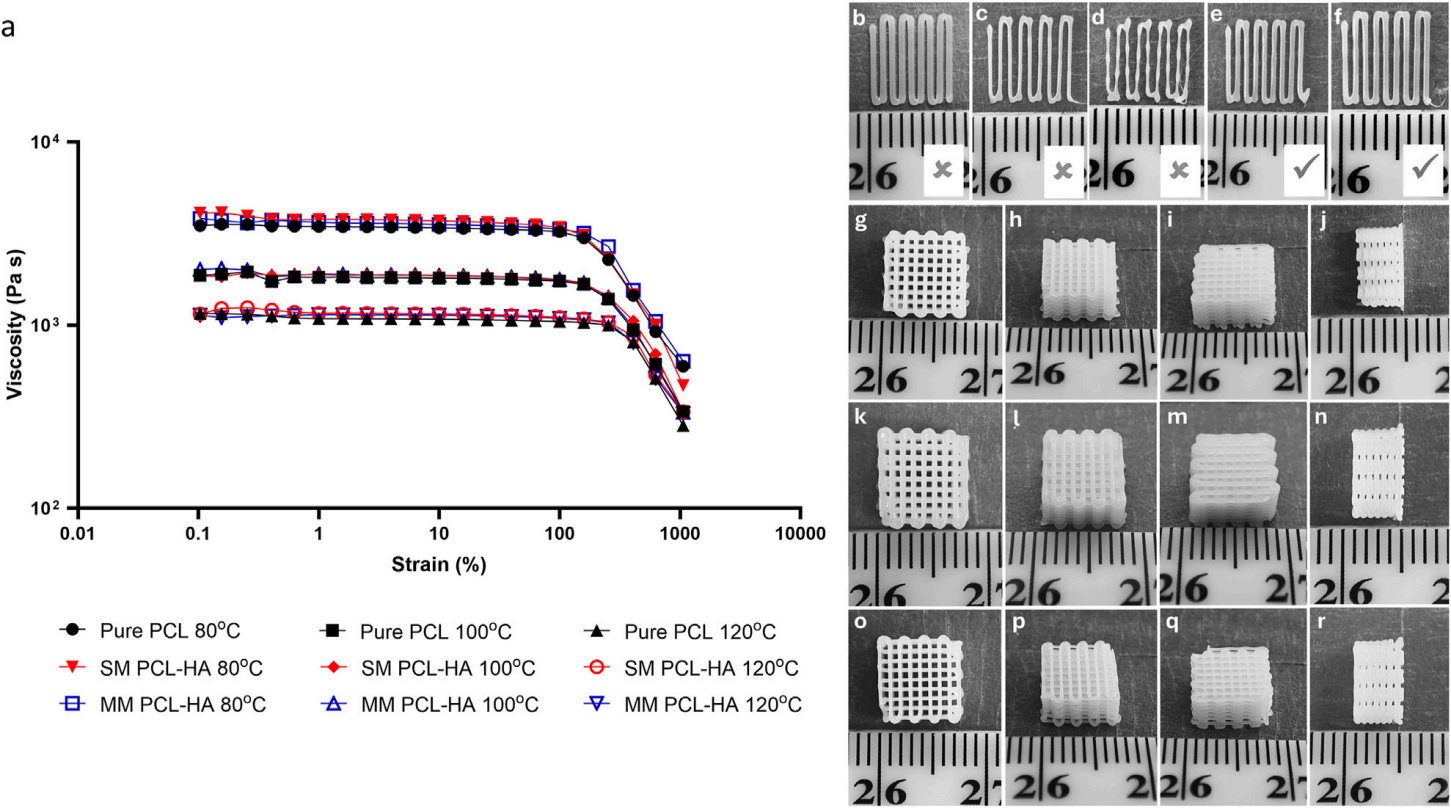
**(A)**: Rheology of PCL-HA composite prepared using solvent method (SM) and melting method (MM) at different temperatures. Pure PCL was used as a control group. **(B–F)**: Visual assessments of printed filament (pure PCL printed at 80°C was used as representatives); **(B)**: excessive pressure (350 kPa); **(C, D)**: insufficient pressure (200 and 150 kPa, respectively); **(E, F)**: optimal pressure (250 and 275 kPa, respectively). **(G–R)**: Representative images of 3D printing results (dimension ∼8 × 8 × 4 mm); **(G–J)**: control (pure PCL); **(K–N)**: solvent method (PCL-HA 90-10); **(O–R)**: melting method (PCL-HA 90-10).

The rheology measurement showed that the difference in ink fabrication method (SM and MM) does not affect the viscosity (Figure 3A). Moreover, adding 10% HA also resulted in negligible changes in the material’s viscosity. In contrast, the increase in temperature had a more significant effect on the material’s viscosity. Furthermore, Figures 3B–R showed evidence of printed filaments during optimisation (Figures 3B–F) and 3D printed scaffolds (Figures 3G–R). Pure PCL was used as a control group in all experiments (n = 4). The optimum printing temperature varied from 80°C to 100°C while the pressure ranged from 175 to 275 kPa (Table 1). Interestingly, although rheological testing indicated that the material’s viscosity did not differ markedly among the groups (Figure 3A), the PCL-HA ink (both SM and MM groups) required a higher temperature than the control group (pure PCL) for 3D printing (Table 1). This may be attributed to the size of the HA particles (<20 μm) and their quantity, which need to pass through a 400 µm nozzle quickly during the 3D printing process; hence, a slight difference in viscosity could be crucial.

**TABLE 1:** Details of optimised printing parameters.

Printing parameter	Control (PCL)	Solvent method (PCL-HA)	Melting method (PCL-HA)
Temperature	80°C	100°C	100°C
Pressure	250–275 kPa	215–275 kPa	175–200 kPa

### 3.2 Physicochemical characterisation of 3D-printed bone scaffolds

#### 3.2.1 Printed scaffold measurement

**FIGURE 4:**
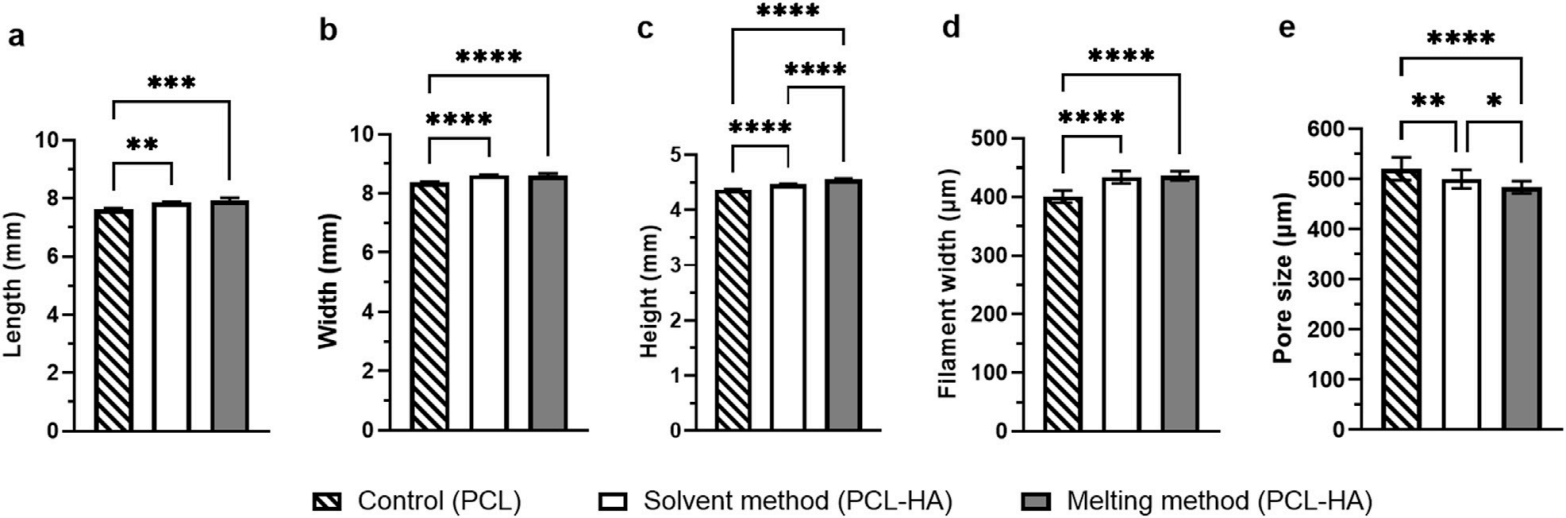
Statistical analysis of scaffold measurements (length **(A)**, width **(B)** and height **(C)** obtained from calliper measurements) and SEM analysis (filament width **(D)** and macropore size **(E)** Note: n = 4; *p<0.05; **p<0.01; ***p<0.001; ****p<0.0001 (analysed using a one-way ANOVA, followed by Tukey *post hoc* test).

All printing methods produced a similar macroscopic appearance in terms of pore size and overall dimensions (Figures 3G–R). The dimension measurement revealed that both the solvent and melting methods could accurately preserve the width and height (Figure 4; Supplementary Appendix S2) compared to the original STL design. Meanwhile, all groups have a decrease in length by ∼0.5–0.8 mm from the original STL design, suggesting 7%–10% shrinkage. However, this offset is very minimal and might not be clinically significant.

#### 3.2.2 Surface morphology analysis using SEM

**FIGURE 5:**
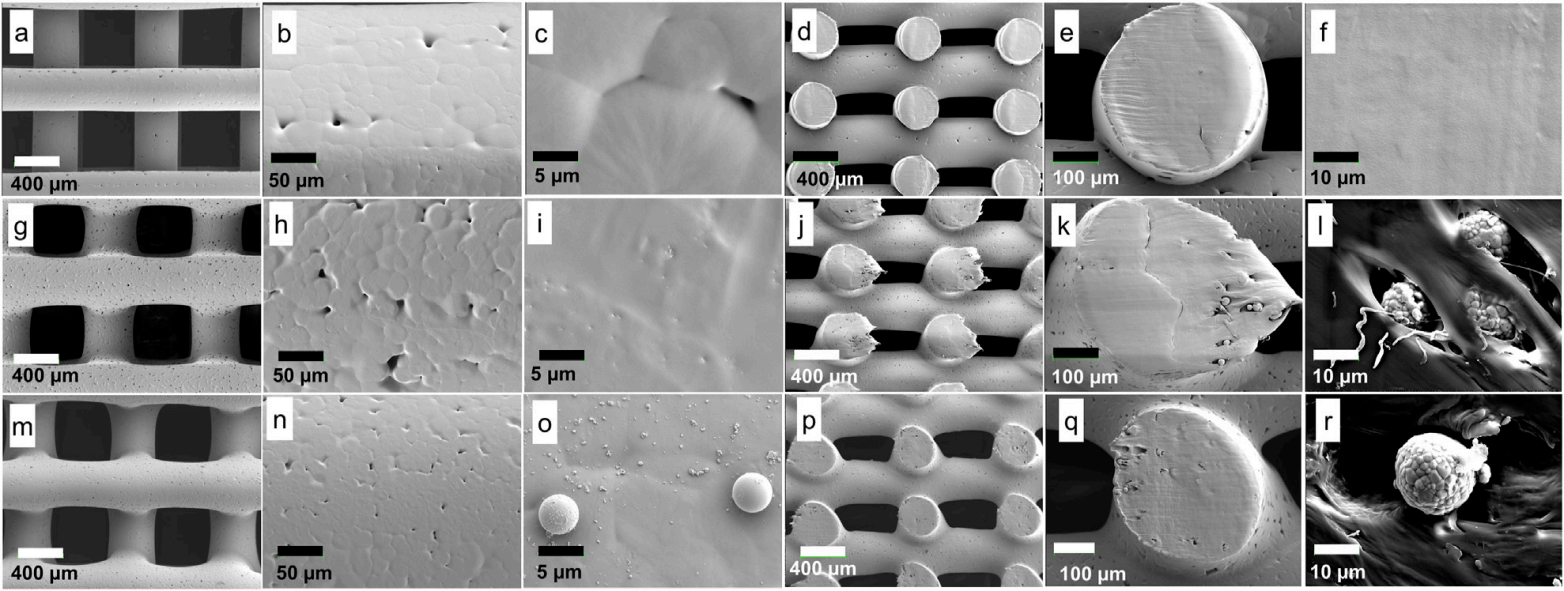
Surface morphology analysis: *Control* **(A–F)**; **(A)**: filaments and macropores; **(B, C)**: filament surface, small micropores were observed (without HA particles); **(D–F)**: vertical cross-section, smooth surface was observed (without HA particles). *Solvent method* **(G–L)**; **(G)**: filaments and macropores; **(H, I)**: filament surface, larger micropores **(H)** and small HA particles **(I)** were observed; **(J–L)**: vertical cross-section, HA particles were observed. *Melting method* **(M–R)**; **(M)**: filaments and macropores; **(N, O)**: filament surface, smaller micropores **(N)** and various sizes of HA particles were observed; **(P–R)**: vertical cross-section, HA particles were observed.

The surface morphology of the control and melting method groups generally displayed fewer micropores compared to those from the solvent method (Figures 5A, B, G, H, M, N, respectively). This disparity may arise from differences in the manufacturing process, wherein rigorous stirring (in the solvent method) induces more air bubbles compared to manual stirring (in the melting method) and no stirring (in the control group). Consequently, upon solvent evaporation, these bubbles result in form of micropores. Additionally, at higher magnification, a variety of sizes of HA particles were observed distributed on the surface (Figures 5I, O), while the control group exhibited no HA particles, as anticipated (Figure 5C). In the vertical cross-section view (Figures 5J–L, P–R), HA particles were also detected inside the filament of the two methods. Meanwhile, the control group showed no HA particles (Figures 5D–F).

It was noted that the scaffold dimensions in all groups were similar (Figure 5; Supplementary Appendix S2), affirming the adequacy of printing parameter optimization. Furthermore, filament width measurements revealed that the solvent and melting methods yielded very similar filament widths when printed under their respective optimized printing parameters (434.36 ± 10.61 μm and 436.43 ± 7.78 μm, respectively) compared to the control (401.14 ± 10.48 μm). In contrast, the macropore size of the melting method was the smallest (483.99 ± 12.42 μm) among the three groups (Figure 5; Supplementary Appendix S2).

The ideal scaffold should have a minimum macropore size of 300–400 μm to allow cell migration, osteogenesis, and neovascularization (Turnbull et al., 2018; Loh and Choong, 2013). Recent literature has also suggested that larger macropores, ranging from 800 to 1,200 μm, promote enhanced osteogenesis (Shi et al., 2022; Shi et al., 2021). Meanwhile, micropores smaller than 10 μm may enhance cell-scaffold interactions due to increased surface area (Turnbull et al., 2018; Li et al., 2021; Zhang et al., 2018). Hence, the solvent method theoretically may facilitate elevated levels of osteogenesis due to larger macropores and greater quantities of micropores.

#### 3.2.3 Energy-dispersive X-ray spectroscopy (EDS or EDX)

**FIGURE 6:**
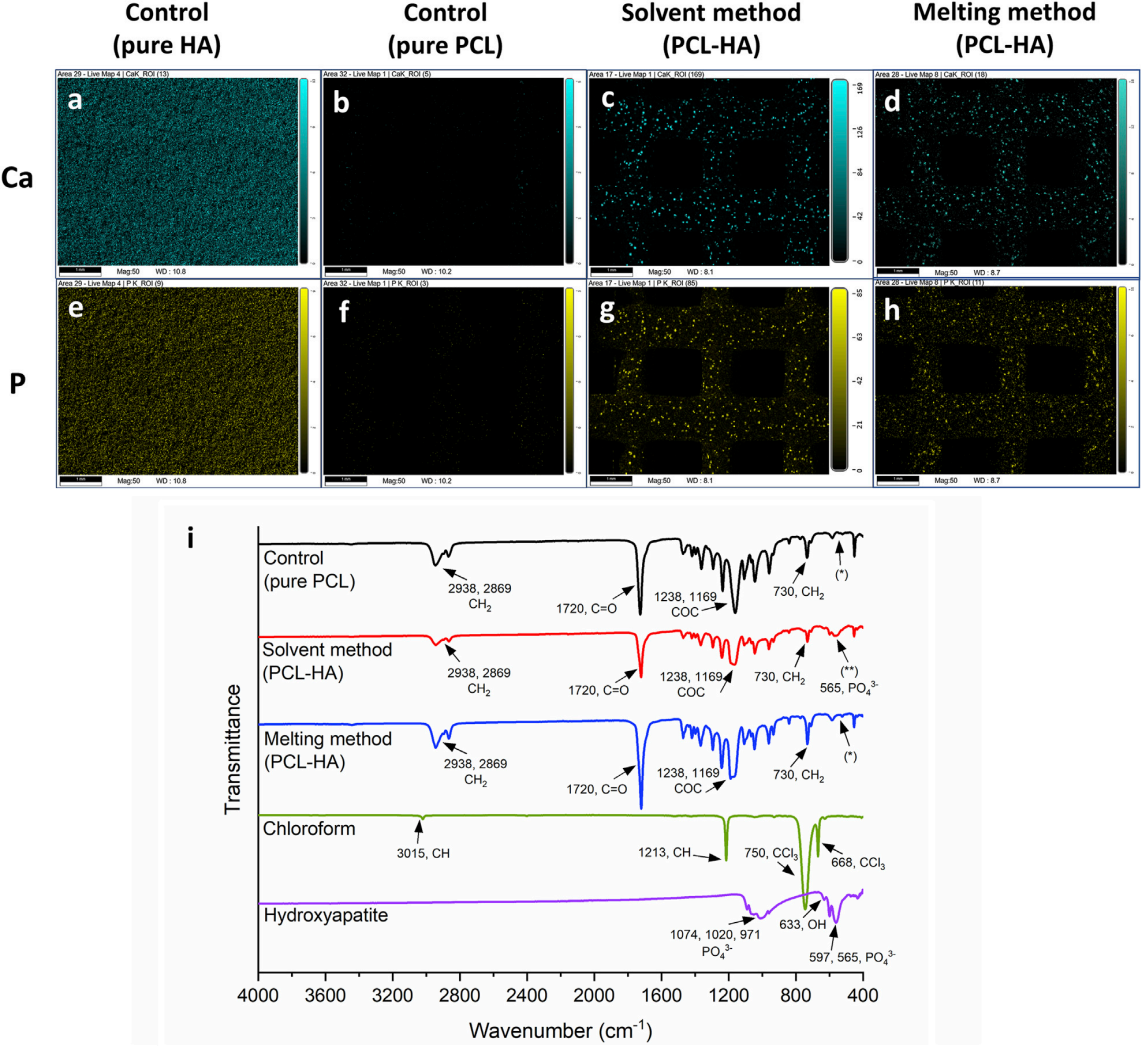
**(A–H)**: Distribution of HA particles across the 3D printed scaffolds by EDX imaging: calcium (blue; **A–D**) and phosphorus (yellow; **E–H**) distributions (scalebar: 1 mm). **(I)**: Fourier Transform Infrared Spectrometer (FTIR) analysis: pure PCL/control group (black line) and pure HA (purple line) spectra were used as a reference (Elzein et al., 2004; Sossa et al., 2018) to detect the change of chemical composition in PCL-HA composite (red and blue lines); chloroform spectrum (green line) was used as a reference (VPL, 2023) to detect the presence of solvent residue in PCL-HA composite fabricated using solvent method (red line). No characteristic peak of HA (565 cm^−1^, phosphate group) was detected in pure PCL/control and MM groups (*), while the presence of HA peak was observed in the SM group (**).

In this study, EDX analysis is performed to visualise HA distribution by detecting its main elements, namely, Ca and P (Figures 6A–H). The analysis shows that HA (represented by Ca and P) was homogenously spread in both methods. No visible difference was observed.

#### 3.2.4 Fourier transform infrared spectroscopy (FTIR)

Figure 6I shows FTIR graphs of PCL-HA composite produced with solvent and melting methods compared to pure PCL (as the control group), pure HA, and chloroform. In Figure 6I, it can be observed that the obtained spectra showed typical peaks for pure PCL (black line), namely, CH_2_ stretching (2,938 and 2,869 cm^−1^), C=O stretching (1720 cm^−1^), C-O-C stretching (1,238 and 1,169 cm^−1^), and CH_2_ rocking (730 cm^−1^) (Tao et al., 2020; Erdal et al., 2020; Elzein et al., 2004).

As references, pure HA and chloroform spectra were also investigated. A characteristic of HA bands was shown in Figure 6I (purple line), specifically PO_4_^3-^ stretching (1,074, 1,020 and 971 cm^-1^), OH^−^ bending (633 cm^−1^), and PO_4_^3−^ bending (597 and 565 cm^−1^) (Sossa et al., 2018). Likewise, our measurement of chloroform spectra (green line) was also typical, i.e., CH stretching (3,015 cm^−1^), CH bending (1,213 cm^−1^), and CCl_3_ stretching (750 and 668 cm^−1^) (VPL, 2023).

Interestingly, our analyses reveal that the typical HA spectra peak (565 cm^−1^, phosphate group) was present in the SM group (Figure 6I, red line, **) but absent in the MM group (Figure 6I, blue line, *). The spectra for the MM group were almost identical to those of the control/pure PCL without HA (Figure 6I, black line, *). We postulate that this difference might be due to several factors, including the variations in sample preparation techniques, agglomeration of HA in MM but better dispersion of HA in SM leading to different signal strengths, and distribution of HA particles within PCL matrix (whether on the surface or embedded), although further investigation is needed.

Moreover, there was a slight intensity change in the PCL-HA composite (both solvent and melting methods) compared to the control group (pure PCL). Nevertheless, all the peaks remained similar (except for the 565 cm^-1^ peak in SM), indicating marginal alterations in PCL chemical composition. In addition, our result confirms that no chloroform residue was detected on the surface of PCL-HA composite fabricated using the solvent method, as no chloroform peaks were observed (Figure 6I, red line).

#### 3.2.5 X-ray diffraction analysis (XRD)

**FIGURE 7:**
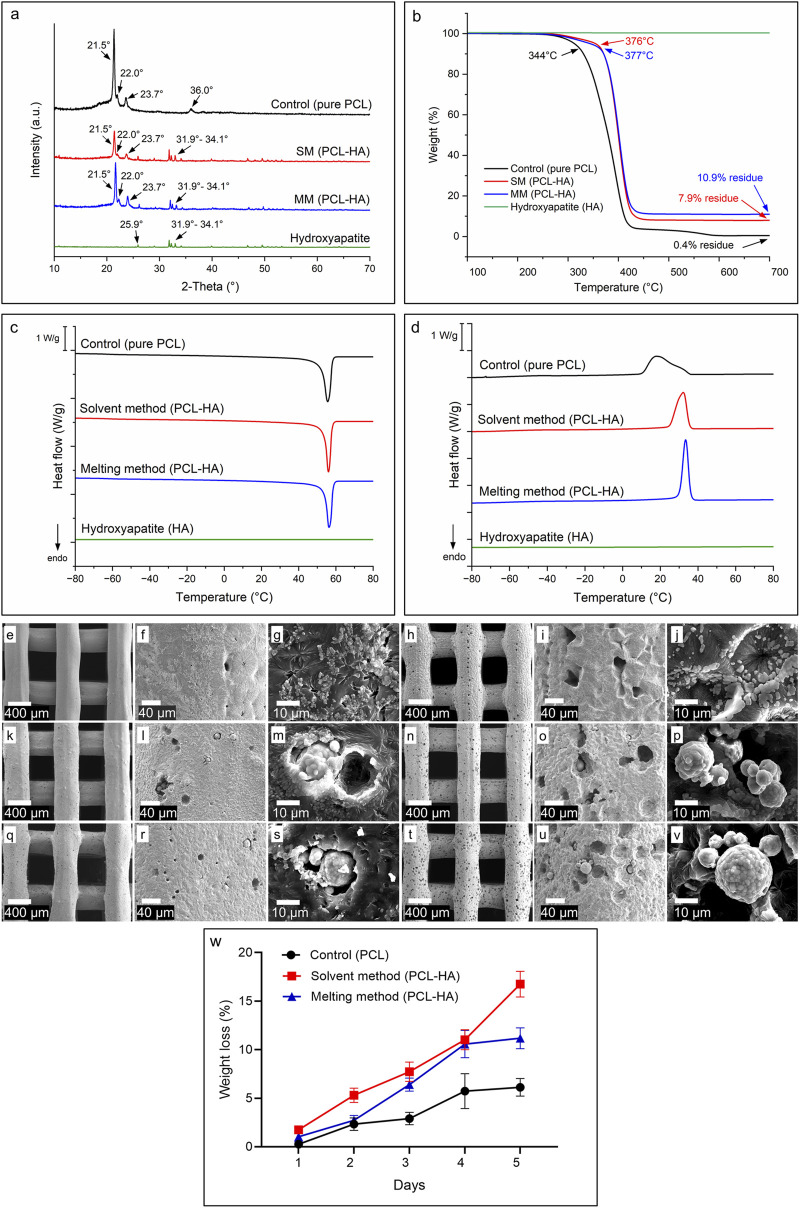
**(A)**: X-ray diffraction analysis (XRD) showing characteristic peaks of PCL, HA, and PCL-HA composite fabricated by solvent and melting methods. **(B)**: Thermogravimetric analysis (TGA) showing degradation temperature and residue of each material. **(C, D)**: Differential scanning calorimetry (DSC) analyses of the materials during heating **(C)** and cooling **(D)** cycles. **(E–V)**: SEM images showing the morphological changes of 3D printed scaffolds due to hydrolytic degradation; control (pure PCL) at day-1 **(E–G)** and day-5 **(H–J)**; solvent method (PCL-HA) at day-1 **(K–M)** and day-5 **(N–P)**; melting method (PCL-HA) at day-1 **(Q–S)** and day-5 **(T–V)**. **(W)**: Weight loss measurement of accelerated degradation test.

The peaks at 2-Theta 21.5°, 22.0° and 23.7° are associated with the [110], [111], and [200] lattice planes of the orthorhombic crystal structure of PCL, whereas the peak at 36.0° corresponds to the [020] plane (Figure 7A). Additionally, hydroxyapatite in the composite materials is identified by a visible peak at 2-Theta of 25.9°, attributed to the [002] plane of HA’s hexagonal lattice, and broad peaks at 31.9°, 32.2°, 33.0°, and 34.1°, which represent the [211], [112], [300] and [202] planes of hydroxyapatite, respectively (Cestari et al., 2021). The XRD results showed that the SM and MM groups preserved the respective PCL and HA characteristics. Moreover, we observed differences in crystallinity as reflected in terms of intensity, as compared to the control group preserving maximum crystallinity and the SM group showing a major amorphous nature (Control > MM > SM). This indicates that both the addition of HA (Control/pure PCL vs. PCL-HA composite in SM and MM) and differences in processing methods (SM vs. MM) influenced the crystallinity of the composite material.

#### 3.2.6 Thermogravimetric analysis (TGA)

The final HA filler content in each PCL-HA composite was determined using TGA, as shown in Figure 7B. The HA content was calculated as the residual mass at 700°C for the SM and MM groups (PCL-HA composites) minus the residual mass of pure PCL (control). The resulting HA concentrations were 7.5 and 10.5 w/w % for the SM and MM groups, respectively. The lower HA content in the SM group, compared to the target value of 10 w/w %, is likely due to the solvent method workflow, which involves pouring and casting the solution into a petri dish. During this process, some proportion of solid HA particles may remain in the walls of the beaker, reducing the final HA concentration in the petri dish. This outcome aligns with findings in the literature on composite preparation using the solvent method (Cestari et al., 2021; Trakoolwannachai et al., 2019) and highlights a potential limitation of this technique.

TGA results also indicated that thermal degradation begins above 340°C for all materials, suggesting that neither the 3D printing process (conducted at 80°C–100°C) nor the composite preparation via the melting method (HA mixed at 180°C) is likely to cause material degradation. The degradation onset temperatures, shown in Figure 7B, are approximately 344°C for pure PCL (control) and 376°C and 377°C for the SM and MM groups, respectively. HA, as expected, remains stable within the observed temperature range.

#### 3.2.7 Differential scanning calorimetry (DSC)

Information about the materials’ properties were determined using DSC (Figures 7C, D). By subjecting the materials to heating and cooling cycles at a constant rate of 10°C min^−1^ between −80°C and 80°C, the glass transition temperatures, melting and crystallisation temperatures, and enthalpies could be obtained (Table 2; Supplementary Appendix S3). The glass transition temperatures and endothermic peaks associated with the polymer melts were similar for all three materials; however, more significant differences became apparent when comparing the different cooling curves. While the onset of crystallisation temperatures for all three materials were similar, the crystallisation peak shapes differed significantly between samples. The crystallisation peak for the melting method was sharp, extending only over 10°C. In contrast, the exothermic crystallisation peak of the control PCL was bimodal and much broader, extending over nearly 40°C (Figure 7D). The solvent method sample, once again showed a more defined crystallisation peak, albeit slightly broader than that of the melting method sample. Despite the differences in peak width, the associated enthalpy values for both the solvent and melting method were similar, while for the control group, the enthalpies were consistently higher. The HA sample showed no enthalpic transitions within the studied temperature range.

**TABLE 2:** Summary of the glass transition temperatures (T_g_), onset temperature of the melt (T_m_), onset temperature of crystallisation (T_c_), melting enthalpy (ΔH_m_) and crystallisation enthalpy (ΔH_c_), extracted from the DSC curves.

Sample	T_g_ (°C)	T_m_ (°C)	ΔH_m_ (J g^−1^)	T_c_ (°C)	ΔH_c_ (J g^−1^)
Control (pure PCL)	−65	52.3	58.5	35.8	59.5
Solvent method (PCL-HA)	−65	53.8	51.9	35.4	52.3
Melting method (PCL-HA)	−65	53.9	51.1	36.1	52.4

Our findings are consistent with the literature (Jiao et al., 2019). As expected, there are some alterations of PCL-HA thermal characteristics due to the addition of HA, with some changes in the onset temperature of the melt (T_m_), onset temperature of crystallisation (T_c_), melting enthalpy (ΔH_m_) and crystallisation enthalpy (ΔH_c_) compared to pure PCL (control group). Nevertheless, the solvent and melting methods both showed almost identical thermal characteristics (Table 2), which points out minimum differences between the two methods (Biscaia et al., 2022).

#### 3.2.8 Accelerated degradation test

Under human physiological conditions, PCL degrades primarily via hydrolysis process, occurring as either non-enzymatic (abiotic) or enzyme-promoted hydrolytic degradation (hereafter addressed as hydrolytic and enzymatic degradation, respectively). Although the enzymatic degradation of PCL is faster than hydrolytic degradation (Castilla-Cortázar et al., 2012), the enzymes involved in PCL degradation are either irrelevant to humans or orthopaedic applications. These enzymes include cutinase (that is present in fungi and bacteria, not humans), lipase (present in the human gastrointestinal tracts—pancreas, mouth, and stomach—but not bones) and esterase enzymes (primarily found in human liver, gastrointestinal tract, skin, and a small amount in blood plasma). While blood plasma is in contact with bone, the low concentration and the specific role of esterase enzyme in PCL degradation at the bone interface remains uncertain and is not the focus of this study (Tajvar et al., 2023; Bartnikowski et al., 2019; Banerjee et al., 2014). Thus, this study emphasizes PCL’s hydrolytic degradation (non-enzymatic) and its relevance to orthopaedic applications.

Due to PCL’s low hydrophilicity, its hydrolytic degradation *in vivo* is slow (up to 2–3 years) (Lam et al., 2009; Sun et al., 2006). On the other hand, accelerated degradation under acidic or basic conditions can mimic physiological conditions within a shorter timeframe, (Huang et al., 2022; Daskalakis et al., 2023; Ang et al., 2007), offering a more practical representative simulation than enzymatic models (Bartnikowski et al., 2019), although verification against long-term data is essential. PCL hydrolytic degradation proceeds through: [1] random hydrolytic chain scission of PCL in its amorphous regions, as those regions were more susceptible to the hydrolytic attacks than the densely packed crystalline regions; [2] ester bond cleavage within the polymer backbone, leading to the formation of smaller oligomers and monomers; and [3] surface and bulk erosion, leading to mass loss and reduced mechanical strength (Tajvar et al., 2023; Bartnikowski et al., 2019). Finally, the degradation end products (CO_2_ and H_2_O) are totally eliminated from the body.

We conducted accelerated degradation tests by immersing PCL in 5 M NaOH to simulate this mechanism. On day 1, both SM and MM scaffolds degraded at similar rates, showing minimal but noticeable changes in surface morphology, such as a coarser texture and the formation of more micropores (Figures 7E–G, K–M, Q–S). By day 5, the SM and MM groups exhibited greater exposure of hydroxyapatite, along with an increase in both the number and size of micropores compared to the control group (Figures 7H–J, N–P, T–V). Based on weight loss measurements, it became evident that the SM group degraded faster than the MM group by day 5, while the control group experienced the slowest degradation rate (Figure 7W). Overall, all groups showed changes in surface morphology and topography during hydrolytic degradation, with the formation of micropores that would support space availability for newly formed bone and exposure of hydroxyapatite within the PCL matrix that potentially benefits osteogenesis.

#### 3.2.9 Mechanical test

**FIGURE 8:**
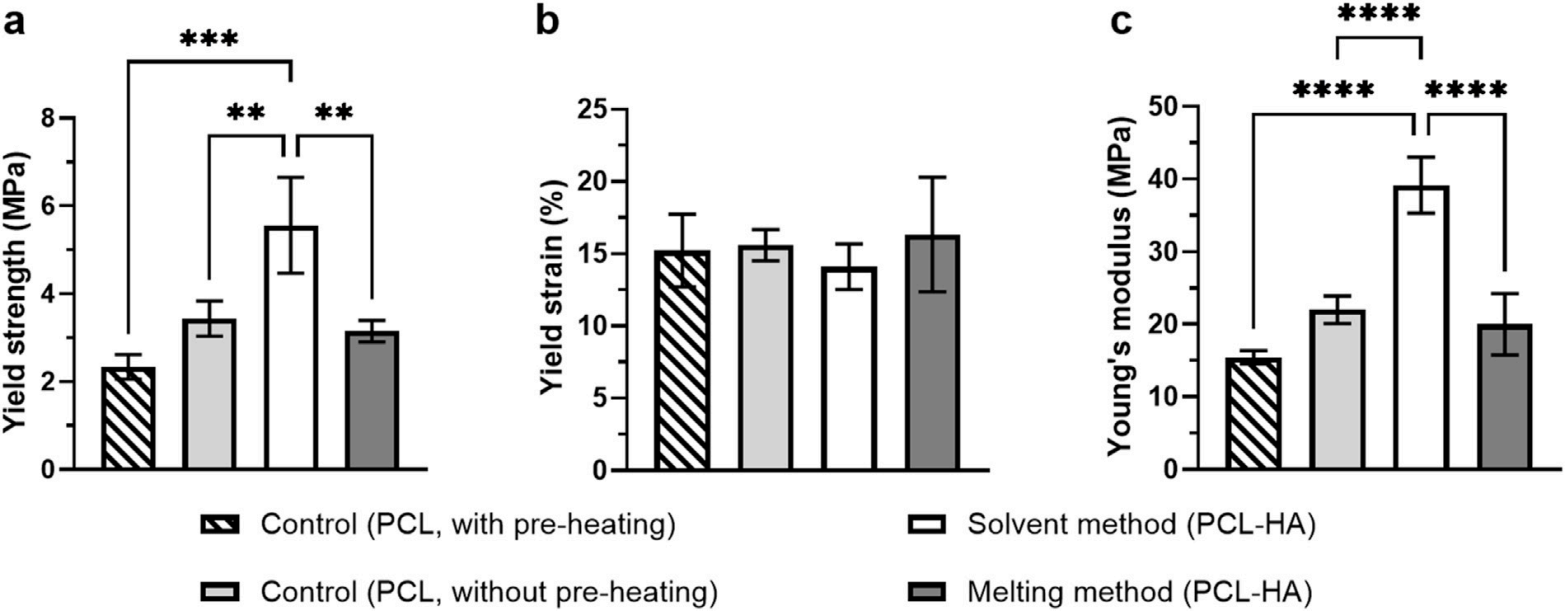
Compressive testing of 3D printed scaffolds: Yield strength **(A)**, yield strain **(B)**, and Young’s modulus **(C)** of the scaffolds (n = 4 = Note: **p<0.01; ***p<0.001; ****p<0.0001 [**(A)** and **(C)** were analysed using one-way ANOVA, while **(B)** was analysed using Kruskal-Wallis].

For compression tests, control samples were categorised into two groups: the control group with pre-heating (in which PCL granules were pre-heated up to 180°C before being printed at 80°C) and without pre-heating (in which PCL granules were directly printed at 80°C). This was performed to investigate if exposure to a high temperature in the melting method (when mixing HA at 180°C) would alter its mechanical properties. Our study revealed that the solvent method yielded the highest mechanical property (Young’s modulus 39.16 ± 3.85 MPa) as compared to other groups. Moreover, exposure to a high temperature of up to 180°C compromised the scaffold’s mechanical properties, as observed from the compression moduli of both control groups (with pre-heating 15.44 ± 0.92 MPa vs. without pre-heating 22.02 ± 1.90 MPa) (Figure 8).

The present findings are also in close agreement with the current literature, indicating that the addition of HA enhanced mechanical properties of 3D printed scaffolds (Cestari et al., 2021; Rezania et al., 2022), as reflected in both PCL-HA groups (solvent and melting methods) compared to the control group (with pre-heating). This is most likely due to the role of fillers such as hydroxyapatite as nucleating agents of polymer, leading to higher crystallinity (Patlolla et al., 2010; Pedrosa et al., 2021). Nevertheless, the 3D printing process involves controlled melting and cooling cycles, which can influence polymer crystallinity and chain orientation in complex ways. Consequently, printed structures may exhibit mechanical properties that differ from those of bulk materials.

Interestingly, despite HA addition, the melting method showed a lower compressive modulus than the control (without pre-heating) (Figure 8). This suggests that high-temperature exposure during HA mixing at 180°C (in the MM group) could negatively affect the scaffold’s mechanical properties, although a more comprehensive investigation is needed. Moreover, although printing at a higher temperature increases the cohesive strength amongst the fused filaments (i.e., bonding capacity), it will also decrease the mechanical strength of the individual filaments (resulting in filament breakage) (Koch et al., 2022) due to the loosening of polymer chains. Hence, the overall structure’s mechanical properties may decrease.

#### 3.2.10 Wettability (contact angle) test

**FIGURE 9:**
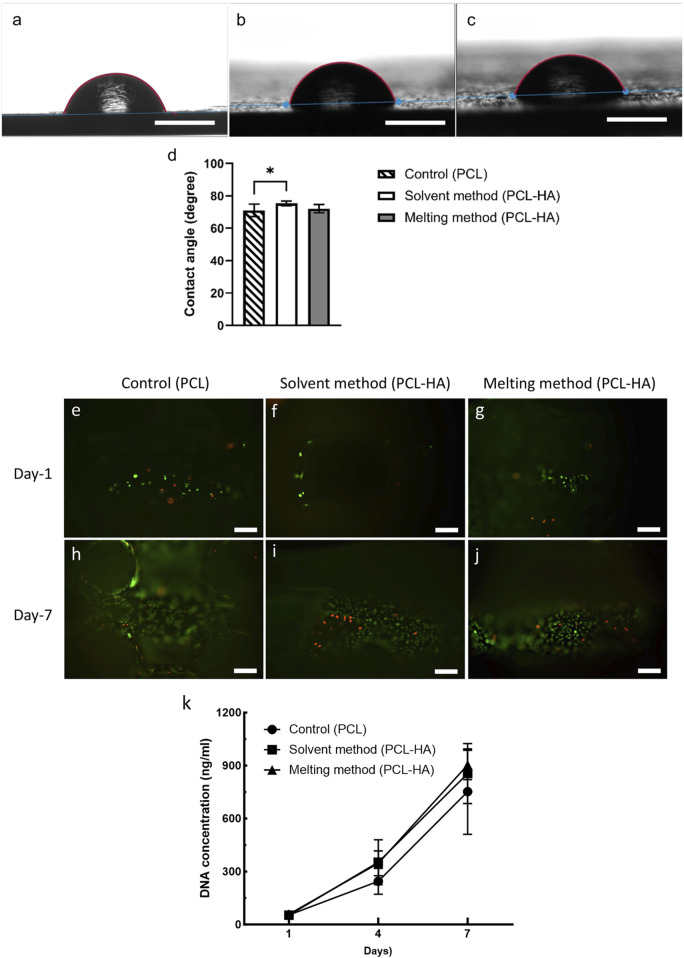
**(A–C)**: Wettability/contact angle test [**(A)**: pure PCL; **(B)**: solvent method; **(C)**: melting method, scalebar: 1 mm]. **(D)**:Statistical analysis of the contact angle measurements (n = 4); *p = 0.015 (analysed using one-way ANOVA). **(E–J)**: Live/dead assay at day-1 **(E–G)** and day-7 **(H–J)** of control group **(E, H)**, solvent method **(F, I)**, and melting method **(G, J)**, scalebar: 100 µm). **(K)**: DNA quantification using Picogreen assay at days 1, 4, and 7 (n = 4).

Wettability measures how well a liquid can spread or adhere to a solid surface. Wettability is measured by the contact angle (CA) test, in which a liquid droplet is placed on the tested biomaterial surface, and the angle formed between the liquid-air interface with the biomaterial surface is measured. Generally, a hydrophilic material corresponds to low CA (<90°) and has a strong wetting capacity, while a hydrophobic material has a high CA (≥90°) with poor wetting capacity (Law, 2014). Although it was long assumed that low CA is better for cell attachment, an extremely hydrophilic material (CA <35°–40°) showed a reduced cell growth than moderately wettable material (40°–60°) (Arima and Iwata, 2007). Our study shows that all tested materials fall into the range of hydrophilic material (control 71.02° ± 3.89°, solvent method 75.36° ± 1.55°, melting method 72.08° ± 2.54°) with minimal differences among the three groups (Figures 9A–D).

### 3.3 Cytocompatibility characterisation of 3D-printed bone scaffold

#### 3.3.1 Live/dead assay

The short-term (7 days) cytocompatibility test using live/dead assay showed that both solvent and melting methods produced cytocompatibility scaffolds (Figures 9E–J). More live cells (green) were observed as compared to dead cells (red) in all scaffolds. The proportion of live and dead cells across the three groups was also found to be comparable.

#### 3.3.2 DNA quantification

We utilised Picogreen assay to quantify the amount of DNA in the samples to measure cell growth. In general, the mean DNA concentration of PCL-HA groups (both solvent and melting methods) were slightly higher than control at day-7 (analysed using two-way ANOVA). Moreover, we observed that the increase of DNA content was the highest in the solvent method, namely, a 6.7-fold and 16.2-fold increase on day-4 and day-7 compared to day-1, inferring higher cell proliferation than the other groups (Figure 9K; Supplementary Appendix S4). However, no significant difference was observed amongst the three groups.

The higher cell proliferation rate in the solvent method might be due to increased microporosity and surface roughness that provides more protein adsorption sites, as well as the role of hydroxyapatite as a biocompatible material that enhances cell adhesion (Li et al., 2021; Deligianni et al., 2001; Shi et al., 2014; Boyan et al., 2017; Liu et al., 2021; Han et al., 2022). However, the insignificant increase implies that the difference in composite preparation method has minor effects on scaffolds’ biological performance. Both solvent and melting methods support cell proliferation and produce cytocompatible scaffolds.

The main finding of this study is summarised in Table 3. We observed that there was not a notable difference in the quality of end products between the two composite preparation methods, except for higher mechanical property (SM showed better mechanical property than MM) and degradation (SM degraded faster than MM). In terms of workflows, we discovered that solvent method has a longer processing time but might be more cost-effective than melting method.

**TABLE 3:** Key findings of this study.

Parameters	Key finding(s)
Rheology	Both methods produced composite with similar viscosity
Visual assessment of printing results	Both methods maintained filament integrity and preserved overall scaffold dimension (length, width, height) compared to the original computer aided drafted design
SEM analysis	• Micropores were more frequent in solvent method. • Both methods showed various sizes of HA particles on the surface and inside the filaments.
Filament width and pore size (macropores) measurement	• Both methods showed similar filament width measurements (∼430 μm) • Pore size (macropores) was slightly larger in solvent method (500 vs. 480 μm)
FTIR	Solvent method using chloroform did not leave any residue.
EDX -imaging	Both methods produced homogenous distribution of HA particles.
TGA	Solvent method results in lower HA residue, showing the loss of solid filler/content during the process, whereas the melting method maintains the HA % almost similar to the initial target. The composites obtained from both methods do not degrade during 3D printing process
DSC	Both the solvent and melting methods display very similar materials properties – glass transition temperatures, melting and crystallisation temperatures, as well as the associated enthalpies
XRD	Solvent method resulted in lower crystallinity than melting method
Compression test	Solvent method resulted in higher Young’s modulus (2-fold higher)
Wettability/contact angle	Both methods had similar wettability (hydrophilic range)
Accelerated degradation	Solvent method facilitated faster degradation than melting method
Live/dead assay	Both methods produced cytocompatible materials with similar cell survival rate
Picogreen assay	Both methods supported cell proliferation (solvent method is slightly better)
Workflow, scalability	Melting method has a more straightforward workflow, whereas solvent method might be more scalable (semi-automated workflow)

## 4 Discussion

While several studies have investigated solvents for electrospinning (Zhang et al., 2019; Altun et al., 2022; Abdal-hay et al., 2018), 3D printing often relies on the liquid solvent method, which is known for its limitations (Huang et al., 2022; Gharibshahian et al., 2023; Chen et al., 2020). These limitations include poor shape retention, residual solvent issues, and weaker mechanical properties compared to melt-blending. Notably, polycaprolactone (PCL)-based 3D printing primarily uses the liquid solvent method with organic solvents such as dichloromethane (DCM) (Zimmerling et al., 2021; Huang et al., 2022; Chou et al., 2021; Zhang et al., 2021; Gogoi et al., 2024), with minimal exploration of alternative solvents. While DCM dissolves PCL effectively, DCM-printed scaffolds exhibit poor strength, rougher texture, faster degradation, and quicker stress relaxation as compared to melt-blending. These limitations may hinder its usefulness in 3D printed bone scaffolds (Huang et al., 2022).

Thus, we explored the solid solvent method (rather than the liquid solvent method) and compared it to the melting method. Nevertheless, solvent selection within the solid solvent method plays a crucial role in determining final scaffold properties. Biscaia et al. (2022) observed that using dimethylformamide (DMF) in the solid solvent method resulted in scaffolds with weaker mechanical properties compared to the melt-blending method (41.59 ± 1.31 vs. 81.01 ± 1.59 MPa, respectively, for PCL-HA 20%) (Biscaia et al., 2022). Conversely, this study using chloroform in the solid solvent method achieved scaffolds with higher mechanical strength than melt-blending (39.16 ± 3.85 vs. 19.99 ± 4.22, respectively, for PCL-HA 10%). Although there are several differences between our study and Biscaia et al., such as different scaffold dimension (L × W × H ≡ 8 × 8 × 16 mm vs. cylindrical scaffolds d = 10 mm h = 2.5 mm), melting temperature (180°C vs. 100°C), and PCL-HA composition (10% vs. 20%), this finding supports the influence of solvent choice on the physicochemical properties of the composites, which ultimately impact cellular behaviour for bone scaffolding.

Several factors contribute to these outcomes, including solvent evaporation rate and the solvent-polymer interaction. These are crucial for ensuring scaffold uniformity and consistent polymer surface properties (Choudhury et al., 2015; Kim et al., 2002; Robertson et al., 2003; Guan et al., 2006). Additionally, solvent selection impacts particle dispersion within the polymer matrix, further influencing the composite structure, morphology, and properties, ultimately affecting cellular responses and tissue regeneration.

Moreover, the most significant drawback of using DMF is its slow evaporation rate, which poses challenges for streamlining workflows, particularly in industrial applications. Hence, this study explored the solid solvent method using chloroform due to its faster evaporation rate. Research by Oliveira et al. (2014) demonstrated 70% chloroform evaporation within 100 min, and our experiments confirmed complete evaporation within 24 h under a fume hood (Oliveira et al., 2014). Supporting this approach, Altun et al. (2022) also reported extended evaporation times with DMF and suggested highly volatile solvents like chloroform for improved efficiency (Altun et al., 2022). Additionally, Choudhury et al. (2015) highlighted chloroform’s advantage in creating bone scaffolds with higher porosity, a desirable quality for bone tissue engineering (Choudhury et al., 2015).

While the solvent method involves more steps (10 steps) and longer processing time (37.6 h) compared to the melt method (4 steps, 13 h), a significant portion of this time is spent on passive tasks like overnight stirring, drying, and storage, requiring minimal intervention. Therefore, the actual hands-on labour difference is negligible (100 min for solvent vs. 60 min for melting method).

The melting method relies on manual stirring on a hot plate, which is labour-intensive and prone to inconsistencies in achieving homogenous blending with HA. Additionally, manual palletization of the composite can introduce inconsistencies, hindering industrial scalability. This necessitates specialized machinery like twin-screw mixers and pellet machines for standardization and automation, although these come at an additional cost (Gkartzou et al., 2017).

In contrast, the solvent method offers greater scalability and lower upfront costs since it utilizes readily available laboratory equipment. The solvent reduces PCL viscosity, allowing for continuous stirring with a standard stirrer. Furthermore, the solvent minimizes HA powder agglomeration and simplifies equipment cleaning, reducing contamination risks. Additionally, the solvent itself is generally more cost-effective compared to specialized machinery.

Our findings indicate minimal differences between the two composite processing methods in producing 3D printed bone scaffolds. As shown in Table 3, both methods resulted in comparable scaffold dimensions, filament width, macropore size, HA particle distribution homogeneity, chemical composition, wettability, and cytocompatibility for both methods. The striking differences between the two methods are the higher mechanical properties of scaffolds produced by the chloroform-based solid solvent method compared to the melting method, along with a more streamlined workflow and better scalability potential. However, the solvent method results in lower final HA % compared to the melting method, which one should also consider when opting for this method. While both methods are viable, the chloroform-based solid solvent approach provides reproducible bone scaffolds with enhanced mechanical properties which is relevant to bone tissue engineering applications.

Another visible difference between the two processing methods is the degradation rate. Our results showed that PCL-HA scaffolds (SM and MM groups) degrade faster as compared to the pure PCL/control group (Figure 7W), which can be attributed to the presence of HA in the matrix. The HA phase disrupts the formation of PCL crystallites, making the composite more amorphous, as further corroborated by the intensity change in XRD results (Figure 7A). Reduced crystallinity enhances the rate of hydrolysis because the ester linkages in the amorphous regions become more vulnerable to water penetration. Consequently, the addition of HA particles accelerates the weight loss of the SM and MM groups and increases their capacity to absorb water during the initial stages of degradation (Ang et al., 2007; Díaz et al., 2014).

Moreover, the difference in crystallinity observed between the SM and MM groups also resulted in different degradation rates. Despite having the same composition (PCL-HA 90–10), the use of chloroform as a solvent in the SM group seems to have affected the chain organization and structure, leading to a less crystalline material (Guarino et al., 2011). The lower XRD intensity in the SM group correlates to a lower crystallinity in the SM as compared to the MM group (Figure 7A), which hence resulted in a higher degradation rate in the SM group (Figure 7W). Thus, a noticeable difference in crystallinity and degradation rate could be clarified as an influence of different material processing methods (SM vs. MM). However, the choice and selection between these two methods should be guided by factors such as the type of application, resources requirement and its cost-effectiveness.

### 4.1 Limitations and future directions

While this work offers a thorough comparison between two popular processing methods for bone scaffold composite fabrication, this study has several limitations. First, we selected a PCL-HA ratio of 90:10 to fabricate bone scaffolds. Although this ratio has been widely used in previous research (Wang et al., 2022; Scocozza et al., 2021; Jiang et al., 2012), it may not represent the optimal PCL-HA composition for bone tissue engineering, as there is a lack of comprehensive comparative studies on different PCL-HA ratios. Investigating the most suitable PCL-HA ratio for bone tissue engineering is beyond the scope of this study, which focuses primarily on examining the differences in composite processing. Future studies should undertake a comprehensive evaluation of various PCL-HA compositions to determine the optimal ratio for bone tissue engineering.

Another limitation is the absence of *in vivo* testing. Comparing the *in vitro* results with *in vivo* data would be valuable for understanding the performance of the scaffolds in a more realistic biological environment. Future research should address this aspect to provide a more complete assessment. Lastly, this study did not examine changes in molecular weight or mechanical properties resulting from PCL degradation, which also merits further investigation in future studies.

## 5 Conclusion

The study conducts a comparative analysis between two prominent methods for fabricating composites for bone scaffolds—the (solid) solvent method and the solvent-free (melting) method. While previous research has explored these methods individually, this study provides a direct comparison of their outcomes in terms of physicochemical properties, cytocompatibility, and mechanical strength. This research highlights a significant finding regarding the mechanical properties and degradation rates of the scaffolds. Contrary to previous assumptions or expectations, the scaffolds produced via the solvent method exhibit superior mechanical strength compared to those obtained from the melting method. The solvent method also facilitated faster degradation rate of the scaffolds compared to the melting method. This novel observation adds a new dimension to the understanding of scaffold fabrication methods and their effects on mechanical performance and degradation rate. The study confirms that both methods demonstrate adequate cytocompatibility and enable a homogenous distribution of hydroxyapatite particles within the scaffolds. This finding reinforces the feasibility of both methods for tissue engineering applications, providing valuable insights for researchers and practitioners. Additionally, the research sheds light on the workflow differences between the two methods and their implications for scalability. By highlighting the labour-intensive nature of the manual stirring process and the variability risk associated with palletisation in the melting method, this study identifies the importance of considering workflow efficiency and automation potential in scaffold fabrication processes.

## Data Availability

The original contributions presented in the study are included in the article/Supplementary Material, further inquiries can be directed to the corresponding author.
