# Supplementary material for: Comparative analysis of solvent-based and solvent-free (melting) methods for fabricating 3D-printed polycaprolactone-hydroxyapatite composite bone scaffolds: physicochemical/mechanical analyses and in vitro cytocompatibility
Source: Front Bioeng Biotechnol. 2025 Jan 6;12:1473777. doi: 10.3389/fbioe.2024.1473777 (PMC11743559; doi:10.3389/fbioe.2024.1473777)
Supplement: Supplementary file 1 [file DataSheet1.pdf]

Supplementary data

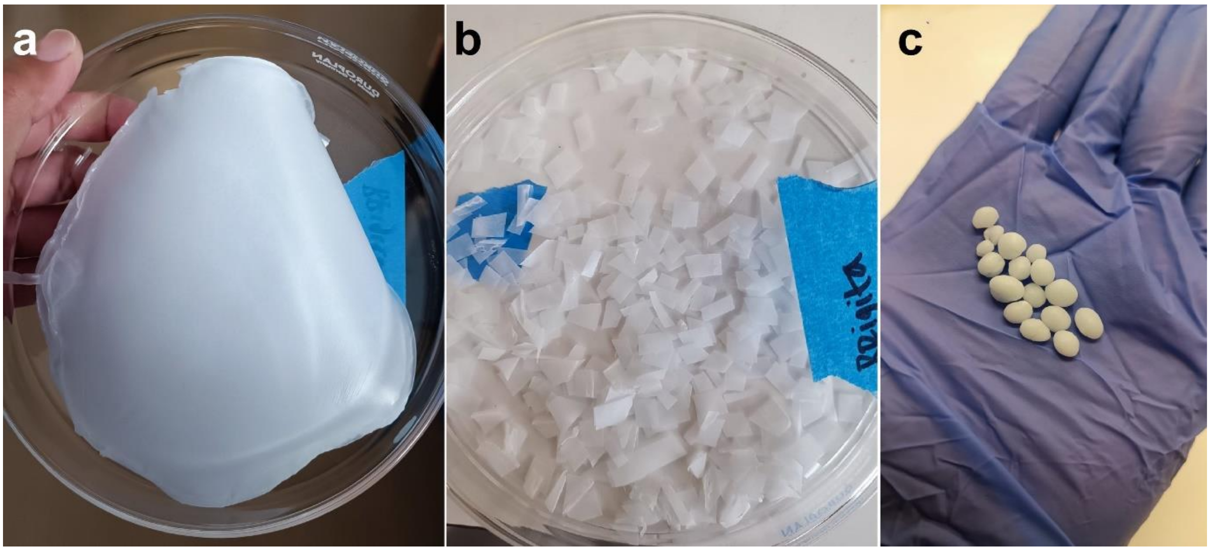

Appendix 1 Results of composite fabrication using solvent and melting method. (a) A sheet of dried PCL-HA casted in a petri dish (solvent method); (b) PCL-HA ink prepared with solvent method was cut into small pieces; (c) PCL-HA ink prepared with melting method was made into small pellets

Appendix 2 Measurement of scaffold dimensions (length, width, height), filament width, and macropore size

**Parameter**	**Control (n=4)**	**Solvent method (n=4)**	**Melting method (n=4)**
**Length (mm)**	7.62 ± 0.04	7.85 ± 0.04	7.92 ± 0.09
**Width (mm)**	8.37 ± 0.03	8.62 ± 0.02	8.61 ± 0.06
**Height (mm)**	4.37 ± 0.01	4.47 ± 0.01	4.55 ± 0.02
**Filament width (µm)**	401.14 ± 10.48	434.36 ± 10.61	436.43 ± 7.78
**Pore size (µm)**	520.74 ± 22.82	500.06 ± 18.55	483.99 ± 12.42

Appendix 3 Differential Scanning Calorimetry (DSC) curves for pure PCL (black), SM group (red) and MM group (blue) at 20 °C min^{-1}. Curves displayed between -80 to -50 °C to highlight the glass transition temperature (T_g) for each sample.

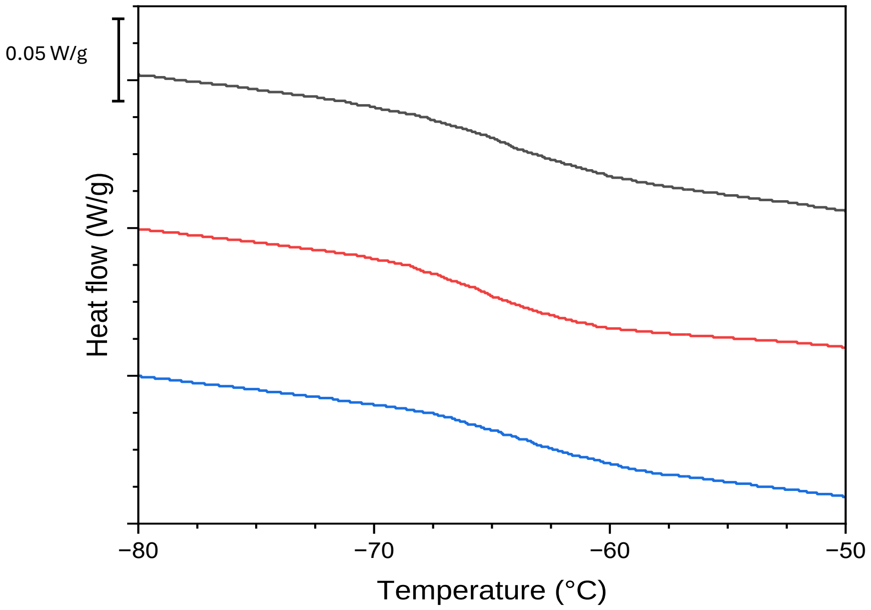

Appendix 4 DNA quantification of cells seeded on the 3D printed scaffolds using Picogreen assay at days 1, 4, and 7 to evaluate cell growth

Groups (n=4)	DNA concentration (ng/ml)			Increase in DNA concentration	
	Day-1	Day-4	Day-7	Day-1 to day-4	Day-1 to day-7
**Control (PCL)**	54.0±9.5	244.5±73.7	752.5±241.4	4.5X	13.9X
**Solvent method (PCL-HA)**	52.7±14.3	352.7±127.3	855.0±170.3	6.7X	16.2X
**Melting method (PCL-HA)**	58.6±11.6	346.7±70.3	903.6±83.5	5.9X	15.4X
